# University of Louisville

**Published:** 1854-09

**Authors:** 


### UNIVERSITY OF LOUISVILLE.

---

THE *preliminary* course of lectures in the Medical Department of the University of Louisville, will commence on the first Monday in October, and as these lectures constitute a virtual extension of the term, it is desirable that they should be attended by students. We assure our readers that the preliminary course is no mere matter of form, but that it will receive the earnest attention of the Faculty. October has been found a season quite as favorable to diligent study as any of the later months of the term, and our impression is, that the lectures delivered during that month are not inferior in interest to those which make up the regular course.

Professor FLINT is expected to return home early in September, and to be in Louisville by the beginning of October, about which time it is the purpose of Professor PALMER to remove to the city.

